# Digital Health Care Industry Ecosystem: Network Analysis

**DOI:** 10.2196/37622

**Published:** 2022-08-17

**Authors:** Yoonseo Park, Sewon Park, Munjae Lee

**Affiliations:** 1 Department of BioMedical Engineering Ajou University Suwon Republic of Korea; 2 Department of Medical Humanities and Social Medicine Ajou University School of Medicine Suwon Republic of Korea

**Keywords:** digital health care, industrial ecosystem, network analysis, topic modeling, South Korea

## Abstract

**Background:**

As the need for digital health care based on mobile devices is increasing, with the rapid development of digital technologies, especially in the face of the COVID-19 pandemic, gaining a better understanding of the industrial structure is needed to activate the use of digital health care.

**Objective:**

The aim of this study was to suggest measures to revitalize the digital health care industry by deriving the stakeholders and major issues with respect to the ecosystem of the industry.

**Methods:**

A total of 1822 newspaper articles were collected using Big Kings, a big data system for news, for a limited period from 2016 to August 2021, when the mobile health care project was promoted in Korea centered on public health centers. The R and NetMiner programs were used for network analysis.

**Results:**

The Korean government and the Ministry of Health and Welfare showed the highest centrality and appeared as major stakeholders, and their common major issues were “reviewing the introduction of telemedicine,” “concerns about bankruptcy of local clinics,” and “building an integrated platform for precision medicine.” In addition, the major stakeholders of medical institutions and companies were Seoul National University Hospital, Kangbuk Samsung Hospital, Ajou University Hospital, Samsung, and Vuno Inc.

**Conclusions:**

This analysis confirmed that the issues related to digital health care are largely composed of telemedicine, data, and health care business. For digital health care to develop as a national innovative growth engine and to be institutionalized, the development of a digital health care fee model that can improve the regulatory system and the cost-effectiveness of patient care, centering on the Ministry of Health and Welfare as a key stakeholder, is essential.

## Introduction

In the 21st century, epidemics, including Severe Acute Respiratory Syndrome and Middle East Respiratory Syndrome, spread rampantly, causing enormous social and economic losses and casualties. Above all else, pandemics such as COVID-19 are directly threatening our health and life, and modern society is entering a situation in which forecasting is more difficult than ever before [1,2]. Due to the prolonged COVID-19 pandemic, the amount of physical activity has decreased by 2%-30%. Measures such as quarantine and social distancing have only highlighted the importance of mental well-being and health care. The disconnection of communication in daily life due to social distancing has a great impact on the happiness and health of individuals. Social and economic factors such as unemployment and loss of income, depression, anxiety, and lack of social communication due to COVID-19 and associated restrictions on physical contact seem to have a negative impact on health. Accordingly, digital health care is being used to compensate for the collapse of the medical system due to the increase in infection and to manage health during disconnection. Digital health care is accessible regardless of location, enabling communication between users. Therefore, it is judged that more digital solutions will be essential not only for health care but also to solve the continuing COVID-19 pandemic and future pandemics.

As digital smart technologies such as smartphones, Internet of Things (IoT), wearable devices, and cloud computing, which were previously outside of the existing medical system area, are rapidly being grafted into the medical field, the utilization of digital health care has been emerging, through which health care can be received anytime and anywhere via various advanced information and communication technologies (ICTs) [3,4]. Digital health care technology is attracting attention as an effective untact (ie, without face-to-face encounters) treatment method in the COVID-19 crisis, which is recognized as an alternative that can be applied to patient evaluation and management [5]. Amid such changes, the medical environment is also expected to undergo a major change; the perception of telemedicine is changing due to the expansion of untact services and treatment caused by self-quarantine [6,7].

Digital health care was first mentioned by Seth R Frank in 2000 [8], which is defined as a service formed by the convergence of the internet and health care, combining health care with the core technologies of the so-called “4th industrial revolution,” including ICT, IoT, cloud computing, big data, and artificial intelligence (AI) [9,10]. In addition, digital health care provides a personalized health management process based on information, including health information, biorhythms, and health behaviors, collected through personal devices and health-related apps [11]. Restricted medical access and other unresolved problems in the medical field have traditionally limited access to medical data; however, a movement has recently emerged to foster the digital health care industry due to the revision of the 3 Data Acts and others [12,13]. A great feature of digital health care is that it enables the treatment and prediction of diseases by utilizing patient big data. Toward this end, it is necessary to effectively utilize big data while maintaining the security of personal information.

In Korea, regulations on data use have prevented digital health care companies from becoming active. However, with revision of the 3 Data Act, big data analysis using pseudonymized data became possible, and this is expected to be a turning point in digital health care innovation using AI and big data. Accordingly, as the development speed of digital technology has become rapidly high and various types of health care services based on mobile devices have been increasing, research is being actively conducted to expand the application of digital health care.

To date, studies on digital health care have mainly focused on user acceptance, intention, and willingness for continuous use. Becker [14] investigated the acceptance intention of mobile health (mHealth) apps, targeting German adults aged 18-35 years, finding that the leakage and loss of personal information in the medical sector is the most sensitive matter to users. In addition, Kim et al [15] analyzed the determinants of the intention to use wearable device products in a middle-aged and elderly population, with the goal of suggesting alternatives to the increase in health care demand and costs due to aging of the population. Moreover, studies on smart health care systems to expand the use of digital health care through big data case studies and providing patient-oriented services are being actively conducted. However, digital health care products involve many stakeholders before they are delivered to users. In the existing health care system, hospitals, pharmaceutical and medical device companies, and patients represent the main stakeholders, whereas the primary stakeholders in the digital health care industry are insurance companies, health care professional services, telecommunication companies, manufacturers of wearable devices (eg, biosensors), and health care app solution providers; thus, a new ecosystem is being formed.

In this changing paradigm, to activate and promote the utilization of digital health care, it is necessary to first understand the industry structure; however, the research in this field remains insufficient. In particular, digital health care products are closely associated with regulations and related policies because they affect the human body. For digital health care products to be practically used, it is necessary to establish future policy directions by grasping the relationship between stakeholders and issues regarding the industry.

Therefore, the aim of this study was to analyze the network of the digital health care industry using newspaper articles that form the basis of social debates on specific issues. Through this approach, we intend to suggest measures to revitalize the industry by deriving the stakeholders and major issues with respect to the ecosystem of the digital health care industry. In other words, by analyzing the ecosystem surrounding the current digital health care industry and identifying key stakeholders and major issues, we can propose policy alternatives for the future development of the industry. The ultimate goal is to provide personalized medical services through digital health care, thereby contributing to reducing social costs through preventive treatment.

## Methods

### Korean Digital Health Care

The major subject of digital health care in South Korea can be classified into a telemedicine pilot project and a mobile health care project. The former started with a remote image diagnosis pilot project as a collaboration between Seoul National University Hospital and Yeoncheon Public Health Center in 1988, which was promoted until the mid-1990s, but could not be activated due to factors such as limitations in ICT, an insufficient socioeconomic environment, and no supporting laws and systems [16]. Subsequently, after revision of the Medical Act to allow telemedicine between doctors and patients in 2003, digital health care has been used for health service accessibility, chronic disease management, and other health care services, centered on both local and public health centers [17]. In the telemedicine pilot that ran from 2014 to 2017, various types of pilot projects were applied for patients with chronic illnesses and visiting nursing systems to offer digital health care services for the disabled, elderly, and other vulnerable groups with access difficulty. A social consensus was not reached due to the risk of misdiagnosis and legal disputes in telemedicine. From this negative perspective, there were limits to the provision of telemedicine, which also limits the use of digital health care. Meanwhile, with the emergence of COVID-19, South Korea has temporarily allowed telemedicine, and has thus been able to provide patient-oriented medical services based on data obtained through digital health care. Through telemedicine, medical services were provided to patients who had difficulty visiting hospitals, improving health service accessibility. In the midst of such changes, even citizens who previously expressed negative views on telemedicine also empathize with the need for telemedicine, resulting in a trend of expansion in the utilization of digital health care in the country [18].

To overcome the limitations of the existing health care service and verify the effectiveness of mHealth, a mobile health care project has been promoted since 2016, centered on public health centers. For this purpose, an mHealth platform was developed, providing health care services by interlocking mobile apps and devices. To meet the demand for preventive health care, customized health care services are provided by utilizing digital health care based on ICT, big data, and other applications in health centers belonging to the public sector. For those with health risk factors for metabolic syndrome, doctors, nurses, nutritionists, exercise specialists, and other professionals are providing customized health counseling for 6 months. Digital health care is being utilized to reduce medical expenses and effectively help national health promotion by continuously managing and preventing chronic diseases and other conditions [19]. However, a health care service delivery system through digital health care has not yet been established owing to limitations in data utilization and related factors. Accordingly, to overcome such limitations and build an effective health management system utilizing digital health care, we performed an analysis on the stakeholders related to the digital health care industry.

### Research Model

**Figure 1:**
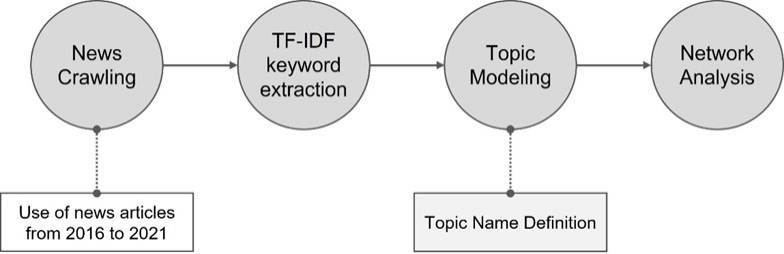
Research model. TF-IDF: term frequency-inverse document frequency.

The purpose of this study was to derive measures to activate digital health care through network analysis. To this end, we used newspaper articles to identify trends in the related industries and analyze associated issues. Newspaper articles reflect public perceptions and industrial opinions on specific topics, enabling broader ecosystem analysis than possible with academic papers. In addition, as newspaper articles can reflect expert opinions, there is an added advantage of including the opinions of more professional stakeholders than possible when analyzing data from social networking sites (SNSs) such as Twitter and Google. The analysis period was set from 2016 to August 2021, when the mobile health care project was promoted, being centered on public health centers. For data collection, we used the news big data system BIG Kinds [20]. BIG Kinds is the largest search engine in Korea, providing articles from 11 central, 8 economic, 28 regional, 5 broadcasting, and 2 specialized magazines. To ensure data reliability, articles were collected using 11 domestic metropolitan newspapers, excluding economic magazines, regional comprehensive magazines, broadcasting companies, and specialized magazines. The search keyword was set to “digital health care,” and a total of 1822 articles were used for analysis. The study flow then followed the order term frequency-inverse document frequency (TF-IDF) keyword extraction, topic modeling, and network analysis (Figure 1).

### Analysis Method

The collected text data were analyzed using programs of R 4.1.1 (RStudio, Inc, Boston, MA, USA) and NetMiner 4.4.1 (Cyram Inc, Seoul, Korea). First, for data preprocessing, we used the “KoNLP” package, a Korean morpheme analysis function of R, and the “koRpus” package, which can extract language samples with a specific purpose for natural language research. Second, TF-IDF weights were extracted from the extracted text, and then major topics were derived using topic modeling. Third, network analysis was performed with NetMiner. Major stakeholders in the digital health care industry and related issues were analyzed through 2-mode network analysis, which can analyze the dual structure of data, such as the relationship between organizations and the relationship between an organization and its members. In other words, the 2-mode network was used because it enabled elucidating the relationships between stakeholders in the digital health care industry, between issues, and between stakeholders and issues.

The TF-IDF weight model is a statistical index to investigate the importance of keywords, which is the weight generated by multiplying the reciprocals of keyword and document frequencies. The larger the TF-IDF value, the more likely it is to determine the meaning or topic of the document to which the relevant word belongs; thus, it is utilized as a measure to extract major keywords from text data [21,22]. TF-IDF analysis is useful for analyzing issues frequently mentioned in SNS or news articles, through which we can discover the main content, information, network relationships, and other aspects regarding the issues of interest [23]. With topic modeling, all keywords included in text are organized by topic, where values within topics are automatically arranged in descending order. Since a large number of keywords are summarized into analysis units referred to as “topics,” the arranged topic can be considered a cluster representing the keywords that constitute the topic. Through this approach, it is possible to understand what topics the keywords are composed of, the importance between keywords, and similar characteristics [24,25]. Since topic modeling has the advantage of considering and identifying all of the multiple topics included in a single document, it has recently become more widely used in studies related to the fields of management, policy, and industry [26,27].

Network analysis is a method used to quantitatively analyze the relationship of individualized nodes, and is also a technique to identify the regular and stylized patterns that are consequently induced by interactions between actors [28]. Network analysis can find various hidden relationship types that are not normally recognized, and can structurally grasp the expression of specific relationship types [29,30]. Network analysis is divided into a 1-mode and 2-mode network according to the analysis target. One-mode network analysis is performed to analyze the relationship between objects when there is an *n* number of objects of the same nature, and the associative relation is analyzed after organizing the objects into an *n*×*n* matrix. When two objects with different properties exist, 2-mode network analysis is used to analyze the relation between them [31].

In addition, to interpret the network analysis, the degree of centrality is measured by using concepts such as the node, link, and connection degree [32]. Centrality is an index expressing the degree to which an actor is centrally located in the entire network; through centrality analysis, it is possible to identify key actors in the network and to determine how close each actor is to the center, along with similar metrics [33]. By showing the position each actor (nodes, keywords) occupies in the overall network and mathematically presenting their size, the actors can be separated into the core part and the periphery of the network. This approach can therefore enable searching for actors playing a central role in the network [34].

There are various types of centrality indices, including degree centrality, betweenness centrality, closeness centrality, and others. In this study, we used degree centrality, which is the most commonly used index in network analysis. Degree centrality refers to the degree of how many neighboring words are connected to a specific keyword; a higher number of connected words indicates higher centrality [35-37]. In degree centrality, the connection degree with other nodes is emphasized, which enables identifying how many relationships the node is involved in [38]. In other words, when using nodes with a high degree of connection, it will be easier to obtain information through their relationships with other nodes. If the nodes disappear in the network, interactions with connected neighboring nodes will be lost, resulting in loss of network function; thus, they are more likely to be key nodes in the network [39,40]. Therefore, in this study, we derived stakeholders and issues regarding the digital health care industry through 2-mode network analysis, also aiming to identify key stakeholders and corresponding major issues with respect to their relationship through evaluation of the degree centrality.

## Results

### TF-IDF Analysis

TF-IDF analysis to identify keywords that have been frequently utilized in the articles analyzed showed that the word with the highest importance among the top 25 keywords (Table 1) was “Medical Care,” indicating that discussions related to the application of digital health care in the medical field have been continuously ongoing. This highest-ranking word was followed by “Government,” “Hospitals,” “COVID-19,” “Care,” and “Insurance,” as the terms with the highest weight values. As a state of public health emergency has been declared in accordance with the spread of COVID-19 and telemedicine services have consequently expanded, it can be seen that the government is promoting precision medicine, smart hospital construction, and so forth. Conversely, keywords with relatively lower weight values were found to be “Regulation,” “Innovation,” “Samsung,” “Seoul National University,” and “Venture” (Table 1). These terms with lower weight demonstrate that discussion is insufficient on the regulatory policy that could be the basis for the activation of digital health care, and that cooperation between companies and universities for product development is not yet active.

**Table 1:** Top 25 ranking keywords based on term frequency-inverse document frequency (TF-IDF) analysis.

Rank	Keywords	TF-IDF
1	Medical care	60.75
2	Government	55.69
3	Hospital	48.76
4	COVID-19	46.52
5	Care	45.61
6	Insurance	44.98
7	Health	42.01
8	Information	40.43
9	Bio	39.92
10	Remote	37.15
11	Data	36.60
12	Company	36.29
13	USA	34.58
14	Seoul	33.84
15	Startup	33.23
16	Technology	31.70
17	Health	31.49
18	Digital	30.22
19	Communication	30.02
20	Economy	27.54
21	Regulation	27.41
22	Innovation	27.13
23	Samsung	26.48
24	Seoul National University	24.31
25	Venture	23.33

### Topic Modeling Analysis

Topic modeling analysis identified a total of 7 topics; we directly assigned the topic name after identifying the correlation between keywords in the topic. Topics 2, 3, and 4 were assigned to “Government”; Topics 1 and 6 were assigned to “Medical Institution”; and Topics 5 and 7 were assigned the topic name “Company.” Keywords belonging to the “Government” topic included Digital, Data, Support, Government, Regulation, COVID-19, and Platform, reflecting the recent movement to invigorate the digital health care industry through the government’s vitalization of the usage of medical data, including the My Data project and the Bio-Health promotion policy. With COVID-19, the demand for untact medical care will further increase continuously in the future; thus, it seems that the necessity to vitalize related industries will be further highlighted. In addition, the government is preparing a foothold for nurturing the digital health care industry via related projects such as establishment of a big data platform, which has limitations in the private capacity.

The keywords belonging to the topic “Medical Institution” included Medical Treatment, Patient, Remote, Smart, Medical Care, and Policy. In the case of medical institutions, it is judged that since the number of outpatients has decreased due to COVID-19, the importance of telemedicine is expanding. In addition, this finding seems to reflect attempts to realize the effective management of patients with chronic diseases and the improvement of health service accessibility by building smart medical care using digital health care products. Conversely, the top keywords belonging to the topic “Company” were Service, Innovation, Device, Startup, Company, and Insurance. In the case of digital health care, those who have access to digital technologies such as AI, ICT, and IoT, even if not belonging to the existing digital health care industry, can readily enter the industry; accordingly, startups are actively being established. Furthermore, to develop digital health care products, cooperation with doctors and hospitals is required; thus, it seems necessary to establish a cooperative company-hospital-doctor system (Table 2).

**Table 2:** Topic modeling analysis.

Themes and topics			Keywords
**Government**			
	Topic 2	Digital, Health, Korea, Field, Support, Provision, Representative, Company, Communication	
	Topic 3	Health, Data, Market, Utilization, Investment, Cooperation, Nurturing, Research, Driving, Change	
	Topic 4	Government, Regulation, COVID-19, Bio, World, Platform, Plan, Individual, Data	
**Medical institution**			
	Topic 1	Government, Patient, Economy, Health, Technology, Medical Treatment, Construction, Cure, Region, Policy	
	Topic 6	Medical Care, Remote, Management, Smart, Seoul, Possibility, Special Zone, Country, Strategy, Apple	
**Company**			
	Topic 5	Service, Innovation, Device, Growth, Future, Startup, Center, Promotion, Diagnosis, Global	
	Topic 7	Industry, Company, Business, Hospital, Insurance, AI^a^, Institution, Doctor, Expansion, Development	

^a^AI: artificial intelligence.

### Network Analysis

**Figure 2:**
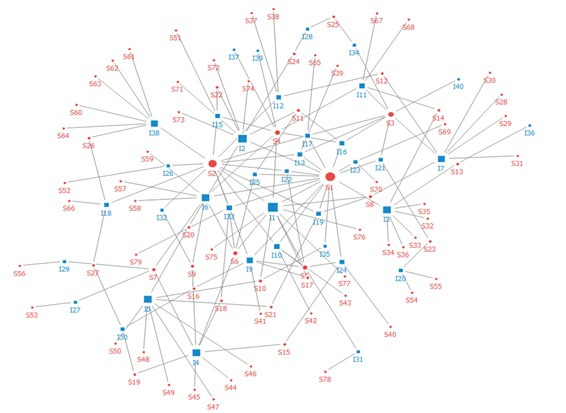
Network analysis. Red indicates stakeholders (circles, S) and blue indicates the main issues (squares, I).

Network analysis was performed to analyze the ecosystem of the digital health care industry, and the relationship between nodes was identified according to the degree centrality (Figure 2). A total of 79 stakeholders and 40 key issues constituted the industrial ecosystem network. The major stakeholders included (1) government and regulatory agencies, represented by the government, Ministry of Health and Welfare, Ministry of Science and ICT, and Regulatory Reform Committee, and (2) medical institutions such as Seoul National University Hospital, Ajou University Hospital, and Gachon University Gil Medical Hospital. Stakeholders also included industrial and maintenance organizations such as the Korea Digital Health Industry Association, Asan Nanum Foundation, Samsung, LG Electronics, and KB Insurance.

The stakeholder showing the greatest centrality was the Ministry of Health and Welfare. In other words, the Ministry of Health and Welfare raises core issues with respect to the network of the digital health care industry ecosystem, representing the actor connected with major issues and other actors in the industry. The government had the second highest centrality value, followed by the Ministry of Food and Drug Safety, Ministry of Science and ICT, and Korean Medical Association in descending order. Thus, the majority of government and regulatory agencies showed higher centrality, except for the three actors ranking in the top 10 with high centrality: the Korean Medical Association, Seoul National University Hospital, and SK Telecom. This seems to be because the digital health care industry ecosystem network became structured, led by the government, on the key issues and actors as the government-centered discussion on the activation of digital health care progressed (Table 3).

Next, we examined the major issues regarding stakeholders. The key issue in the digital health care industry ecosystem network was telemedicine introduction review, followed by passing of the 3 Data Acts, accuracy of AI technology in health care, deregulation of the health care sector, and smart health business (Table 4). Although South Korea has a strong personal information act, the development possibility of telemedicine has increased since revision of the 3 Data Acts was passed in the National Assembly. Particularly, telemedicine services have largely expanded in recent years since the state of public health emergency was declared according to the spread of COVID-19. Our findings further confirmed that deregulation in the health care sector, platform construction, and personnel training in related fields are emerging as major issues due to the expansion of private participation in digital health care. Moreover, other issues have been discussed, such as smart care policy, job creation, and AI real-time prediction of disease diagnosis and infection route (Table 4).

**Table 3:** Centrality of stakeholders.

Rank	Stakeholder	Number of nodes	Centrality
1	Ministry of Health and Welfare	17	0.425
2	Government	14	0.350
3	Ministry of Food and Drug Safety	8	0.200
4	Ministry of Science and ICT^a^	7	0.175
5	Korean Medical Association	6	0.150
6	Ministry of Strategy and Finance	5	0.125
7	Ministry of Trade, Industry and Energy	4	0.100
8	Seoul National University Hospital	3	0.075
9	Prime Minister	3	0.075
10	SK Telecom	3	0.075
11	Health Insurance Review & Assessment Service	3	0.075
12	Samsung	2	0.075
13	Watson Division	2	0.050
14	Vuno Inc	2	0.050
15	Democratic Party of Korea	2	0.050
16	Korea Health Industry Development Institute	2	0.050
17	National Assembly	2	0.050
18	People’s Solidarity for Participatory Democracy	2	0.050
19	Korea Economic Research Institute	2	0.050
20	Korea Insurance Research Institute	2	0.050
21	Kangbuk Samsung Hospital	2	0.050
22	Korea Pharmaceutical and Bio-Pharma Manufacturers Association	2	0.050
23	Osong Medical Innovation Foundation	2	0.050
24	National Health Insurance Corporation	2	0.050
25	Korea Internet & Security Agency	2	0.050
26	Gangwon-do	2	0.050
27	Ministry of SMEs^b^ and Startups	2	0.050
28	LG Electronics	1	0.025
29	Medical Graduate School	1	0.025
30	KT	1	0.025
31	Green Cross Corporation	1	0.025
32	GE Healthcare	1	0.025
33	Ajou University Hospital		0.025
34	AI^c^ Precision Medical Promotion Group	1	0.025
35	Gachon University Gil Medical Center	1	0.025
36	National Human Rights Commission of Korea	1	0.025
37	Apple	1	0.025
38	Regulatory Reform Committee	1	0.025
39	Seoul National University Bundang Hospital	1	0.025
40	Korean Pharmaceutical Association	1	0.025
41	Research Institute for Healthcare Policy under the KMA^d^	1	0.025
42	U Health Industry Headquarters	1	0.025
43	Asan Medical Center	1	0.025
44	The People’s Party	1	0.025
45	Innovation Growth Headquarters	1	0.025
46	Samsung Fire Insurance	1	0.025
47	Kyobo Life Insurance	1	0.025
48	KB Insurance	1	0.025
49	Financial Supervisory Service	1	0.025
50	Financial Services Commission	1	0.025
51	Korea Digital Health Industry Association	1	0.025
52	Public Health Center	1	0.025
53	Korea Institute for Advancement of Technology	1	0.025
54	Siemens	1	0.025
55	KEPCO KDN	1	0.025
56	Korea International Trade Association	1	0.025
57	Electronics and Telecommunications Research Institute	1	0.025
58	Ministry of Public Administration and Security	1	0.025
59	Korea Venture Business Association	1	0.025
60	Minister Park Young-Sun	1	0.025
61	Deliberative Committee of Special Cases on Regulation	1	0.025
62	Korea Venture Investment Corporation	1	0.025
63	Chungcheongbuk-do	1	0.025
64	Busan	1	0.025
65	National Bio-bank of Korea	1	0.025
66	Ulsan	1	0.025
67	National Institute of Food and Drug Safety Evaluation	1	0.025
68	FDA^e^	1	0.025
69	Sejong-si	1	0.025
70	OECD^f^	1	0.025
71	National Cancer Center	1	0.025
72	Biotechnology Industry Organization	1	0.025
73	Korea Centers for Disease Control and Prevention	1	0.025
74	Korea Life Insurance Association	1	0.025
75	Regulatory Reform Committee	1	0.025
76	Seoul St. Mary's Hospital	1	0.025
77	Asan Nanum Foundation	1	0.025
78	Social Welfare Committee	1	0.025
79	Korea Health Promotion Institute	1	0.025

^a^ICT: information and communications technology.

^b^SME: small and medium-sized enterprise.

^c^AI: artificial intelligence.

^d^KMA: Korea Meteorological Administration.

^e^FDA: Food and Drug Association.

^f^OECD: Organisation for Economic Co-operation and Development.

**Table 4:** Centrality of chief issues.

Rank	Chief issues	Number of nodes	Centrality
1	Review of telemedicine introduction	11	0.139
2	3 Data Acts passed	9	0.113
3	Accuracy of health care AI^a^ technology	8	0.101
4	Deregulation in the health care sector	8	0.101
5	Health care insurance products	8	0.101
6	Fostering 5G front-back industries	8	0.101
7	Smart health business	7	0.088
8	Promotion of regulation-free special zones for fostering new industries	7	0.088
9	Concerns over village doctors going bankrupt	6	0.075
10	Establishment of precision medicine–integrated platforms	5	0.063
11	Personnel expansion for permission review	5	0.063
12	Health care platform market	4	0.050
13	Medical information big data	4	0.050
14	Conditional introduction of untact treatment	4	0.050
15	Regulatory sandbox	4	0.050
16	Resolving the personnel shortage of the Ministry of Food and Drug Safety	4	0.050
17	Construction of bio big data	4	0.050
18	Implementation of remote multidisciplinary diagnosis of primary medical institutions	4	0.050
19	Accelerating medical commercialization	4	0.050
20	Silver health care services	3	0.037
21	New drug development support	3	0.037
22	Medical Act amendment	3	0.037
23	Individual-led medical data activation	3	0.037
24	Activation of untact treatment	3	0.037
25	Health insurance fees for telemedicine	3	0.037
26	Judgment of whether the medical treatment is prompt	2	0.025
27	Blueprint of National Innovation Clusters	2	0.025
28	Probability prediction of diabetes and cardiovascular disease	2	0.025
29	Introduction of general digital norms	2	0.025
30	Silver robot expert	2	0.025
31	Need to expand public health services	2	0.025
32	Creation of funds dedicated to the regulation-free special zone	2	0.025
33	Expansion of untact health care services	2	0.025
34	Review for medical device cyber security permission	2	0.025
35	Smart care policy	2	0.025
36	Side effects of AI medical devices	1	0.012
37	Creation of jobs	1	0.012
38	Fostering small- and medium-sized ventures in new industries	1	0.012
39	AI real-time disease diagnosis and infection route prediction	1	0.012
40	Digital medicine	1	0.012

^a^AI: artificial intelligence.

## Discussion

### Principal Findings

As a result of network analysis on the digital health care industry, the government and the Ministry of Health and Welfare of Korea showed the highest centrality and were thus found to be major stakeholders; the major issues they had in common were review of telemedicine introduction, concerns over village doctors going bankrupt, and establishment of precision medicine–integrated platforms. Currently, the government is temporarily allowing telemedicine to respond to the spread of COVID-19, and intends to apply digital health care for remote patient monitoring and disease treatment to cope with insufficient medical demand. In addition, the issue of telemedicine is related to not only government agencies, including the National Assembly and Ministry of Science and ICT; but also medical institutions such as Seoul National University Hospital; and medical industry groups such as SK Telecom, Asan Nanum Foundation, and the Korean Medical Association.

In South Korea, telemedicine has been continuously discussed since the doctor/health care provider pilot project in 2002, but was not institutionalized due to the possibility of misdiagnosis, concerns over village doctors going bankrupt owing to the concentration of patients in large hospitals, and other related issues. As the digital health care industry has developed owing to the development of AI diagnosis technology, popularization of smart devices, and expansion of the utilization of medical data, the possibility of introducing telemedicine has also increased, and citizens’ perception of telemedicine is changing owing to COVID-19. Therefore, the government, industrial sector, and medical care–related organizations should create institutional measures that could support digital health care through continuous discussions on the introduction of telemedicine that can contribute to improving public health. In particular, to prevent the concentration of large hospitals, which is a concern of medical institutions, it seems that patient convenience will improve, which could in turn improve the quality of medical care if primary medical institutions treat chronic diseases. Represcription of patients with mild conditions and patient monitoring for disease prevention could be achieved through the multidisciplinary treatment of primary medical institutions and large hospitals by utilizing digital health care products such as wearable devices. In this regard, an emergency medical system that can provide optimal treatment by collecting vital signs and images of patients in a severe emergency condition using digital health care in real time is being applied to the medical field.

Currently, there are only a few regional trauma and emergency medical centers in South Korea located in county areas, with the majority of such centers located in large cities such as Seoul and Gyeonggi. For emergency and trauma patients, the time required for treatment is important; thus, a balanced arrangement between regions is required. Therefore, digital health care can help to reduce the gap in medical infrastructure between rural and urban areas to better treat and manage patients with severe diseases. Expanding the functions of regional medical institutions by providing appropriate treatment for each emergency patient is expected to lay the foundation for expanding the utility of digital health care.

The major stakeholders of medical institutions and companies were found to be Seoul National University Hospital, Kangbuk Samsung Hospital, Ajou University Hospital, Samsung, and Vuno Inc, and their main issues were accuracy of health care AI technology and smart health business. Safety issues with respect to the utilization of digital health care have been constantly raised. Poor technical accuracy may lead to medical accidents; therefore, institutional strategies must be established in preparation of the possibility of misdiagnosis caused by product defects. It is necessary to improve the performance of digital health care products used for diagnosis and prescription, and to train professional personnel to develop and use the products appropriately. Furthermore, since data measurement must be accurate so as to increase the accuracy of diagnosis, it is judged thereby that periodic education for patients and medical staff in using the product will be necessary. Meanwhile, digital health care is an industry requiring medical information and continuous clinical data; with the increase in the use of digital health care, medical institutions are changing their position from a consumer to a supplier. Accordingly, if digital health care product development is accomplished through industry-university collaboration, it will be possible to develop safe digital health care products through continuously provided data, and in turn establish a smart health care business that can vitalize the industry.

### Conclusion

This analysis confirmed that the major stakeholders in the digital health care industry of South Korea are largely composed of the government, medical institutions, and industrial companies, and that the issues related to digital health care largely consist of telemedicine, data, and health care business. We considered that all government, medical institutions, and industrial companies need to apply digital health care to the medical system through telemedicine and health care business establishment, and that cooperation is necessary among the government, medical institutions, corporations, research institutes, and related stakeholders. For practical cooperation, efficient use of data between institutions is required. Currently, medical data are stored in different ways between institutions, and there is a limit to the use of these data as there is no integrated management. Therefore, it is necessary to enable the use of integrated medical data for the commercialization of digital health care through standardized data linkage. This will not only revitalize the digital health care industry but will also lay the foundation for providing patient-tailored medical services, enabling the realization of precision medicine.

Meanwhile, for digital health care products to be effectively incorporated into the medical system, deregulation and the preparation for health insurance fees are necessary; however, there seems to be insufficient discussion on this aspect. The Ministry of Health and Welfare, a key stakeholder in the digital health care industry, is trying to vitalize the digital health care industry by preparing telemedicine health insurance rates; however, this analysis showed no connection of the Ministry with deregulation in the health care sector, the designation of regulation-free special zones, and other related issues. To vitalize the digital health care industry, it is necessary to expand the usability of patients through deregulation in related fields and application of health insurance fees; however, it seems that there has been no substantial institutional improvement on these aspects, as the discussion has mainly involved only government agencies, civic groups, and interest groups belonging to the related fields. Therefore, to promote the development of digital health care as a national innovative growth engine and its institutionalization, the development of a digital health care fee model that can improve the regulatory system and enhance the cost-effectiveness of patient treatment is essential, which will need to be centered on the Ministry of Health and Welfare as a key stakeholder. To revitalize the digital health care industry, as a national strategic project, the regulatory paradigm should be rationally established and centered on the market economy. This is expected to have a positive impact on the development of related industries by expanding into digital health care–related software, the medical service industry, and the insurance industry in the future, and ultimately enable a preventive-centered medical format through innovative medical service provision.

There are some limitations of this study that should be mentioned. Although newspaper articles have the advantage of providing a large amount of information, including opinions from various stakeholders on a specific topic, they also have a limitation in presenting data reflecting the popular experience on digital health care. Accordingly, future work exploring the issues regarding the digital health care industry by subdividing and analyzing data from SNS such as Twitter, blogs, and Facebook could better reflect the direct experience of digital health care and the views of the government, medical institutions, companies, and general consumers. In addition, we did not perform an analysis of the ecosystem according to the change in digital health care technology. In future research, it will be necessary to prepare a plan to provide digital health care for each patient type by analyzing the ecosystem according to technological change over time, which can help to identify issues and the structure of technology.

## Abbreviations

AI: artificial intelligence
ICT: information and communications technology
IoT: Internet of Things
mHealth: mobile health
SNS: social network site
TF-IDF: term frequency-inverse document frequency

## References

[1] Arendt F, Markiewitz A, Mestas M, Scherr S (2020). COVID-19 pandemic, government responses, and public mental health: Investigating consequences through crisis hotline calls in two countries. Soc Sci Med.

[2] Schwab P, DuMont Schütte A, Dietz B, Bauer S (2020). Clinical predictive models for COVID-19: systematic study. J Med Internet Res.

[3] Yoo B, Jeon H (2021). A study on subjective perceptions of COVID-19 situation of senior living facilities worker. Korea Acad Care Manag.

[4] Lim Y (2021). COVID-19 blues: a big data analysis. J Korean Soc Phys Ther.

[5] Santos-Moreno P, Rodríguez-Vargas GS, Casanova R, Rubio-Rubio J, Chávez-Chávez J, Rivera-Triana D, Castiblanco-Montañez RA, Hernández-Zambrano SM, Villareal L, Rojas-Villarraga A (2021). Evaluation of a non-face-to-face multidisciplinary health care model in a population with rheumatoid arthritis vulnerable to COVID-19 in a health emergency situation. Healthcare.

[6] Jin S (2020). A study of factors affecting use intention of untact medical diagnosis and consultation services. J Korea Contents Assoc.

[7] Lee JS (2020). A study on the activation of telemedicine in COVID-19 pandemic. J Knowl Inf Technol Syst.

[8] Frank SR (2000). Digital health care--the convergence of health care and the internet. J Ambul Care Manage.

[9] Kim KB (2020). A study of the digital healthcare industry in the Fourth Industrial Revolution. J Converg Inf Technol.

[10] Tortorella GL, Fogliatto FS, Saurin TA, Tonetto LM, McFarlane D (2022). Contributions of Healthcare 4.0 digital applications to the resilience of healthcare organizations during the COVID-19 outbreak. Technovation.

[11] Nikou S, Agahari W, Keijzer-Broers W, de Reuver M (2020). Digital healthcare technology adoption by elderly people: a capability approach model. Telemat Informat.

[12] Lee WH (2021). Evaluation and management of dysphagia based on digital health technologies. J Kor Dysphagia Soc.

[13] Oh JH (2021). A study on the perception of Data 3 Act through big data analysis. J Converg Secur.

[14] Becker D (2016). Acceptance of mobile mental health treatment applications. Procedia Comput Sci.

[15] Kim YW, Han S, Kim KS (2018). Determinants of intention to use digital healthcare service of middle and older users. Inf Soc Media.

[16] Lee J (2020). A research on the necessity for telemedicine in the digital healthcare era. Dong A Law Rev.

[17] Lee M, Park S, Lee K (2020). Relationship between morbidity and health behavior in chronic diseases. J Clin Med.

[18] Mobbs RJ, Ho D, Choy WJ, Betteridge C, Lin H (2020). COVID-19 is shifting the adoption of wearable monitoring and telemedicine (WearTel) in the delivery of healthcare: opinion piece. Ann Transl Med.

[19] Yoon K, Park S, Choi S, Lee M (2020). A proposal for public health information system-based health promotion services. Processes.

[20] BIG Kinds.

[21] Chen K, Zhang Z, Long J, Zhang H (2016). Turning from TF-IDF to TF-IGM for term weighting in text classification. Exp Syst Appl.

[22] Kim D, Seo D, Cho S, Kang P (2019). Multi-co-training for document classification using various document representations: TF–IDF, LDA, and Doc2Vec. Inf Sci.

[23] Wang Z, Hahn K, Kim Y, Song S, Seo J (2017). A news-topic recommender system based on keywords extraction. Multimed Tools Appl.

[24] Ahadh A, Binish GV, Srinivasan R (2021). Text mining of accident reports using semi-supervised keyword extraction and topic modeling. Process Safety Env Protect.

[25] Yun H, Park J, Yoon J (2019). Introduction of topic modeling for extracting potential information from unstructured text data: issue analysis on news article of dementia-related physical activity. Korean J Sport Sci.

[26] Fu Q, Zhuang Y, Gu J, Zhu Y, Guo X (2021). Agreeing to disagree: choosing among eight topic-modeling methods. Big Data Res.

[27] Kim SM, Kim Y`j (2020). Research trend analysis on living lab using text mining. J Digit Converg.

[28] Leng Z, Sun H, Cheng J, Wang H, Yao Z (2021). China's rare earth industry technological innovation structure and driving factors: a social network analysis based on patents. Resour Policy.

[29] Shukla M, Wu AF, Lavi I, Riddleston L, Hutchinson T, Lau JY (2022). A network analysis of adolescent mental well-being during the coronavirus pandemic: evidence for cross-cultural differences in central features. Pers Individ Dif.

[30] Taylor S, Landry CA, Paluszek MM, Rachor GS, Asmundson GJ (2020). Worry, avoidance, and coping during the COVID-19 pandemic: a comprehensive network analysis. J Anxiety Disord.

[31] Lee M, Yoon K (2018). Ecosystem of the medical device industry in South Korea: a network analysis approach. Health Policy Technol.

[32] Yuan CT, Nembhard IM, Kane GC (2020). The influence of peer beliefs on nurses' use of new health information technology: a social network analysis. Soc Sci Med.

[33] Alotaibi N, Rhouma D (2021). A review on community structures detection in time evolving social networks. J King Saud Univ Comput Inf Sci.

[34] Lee M, Yoon K, Lee K (2018). Social network analysis in the legislative process in the Korean medical device industry. Inquiry.

[35] Behzadifar M, Gorji HA, Rezapour A, Rezvanian A, Bragazzi NL, Vatankhah S (2019). Hepatitis C virus-related policy-making in Iran: a stakeholder and social network analysis. Health Res Policy Syst.

[36] Li S, Garces E, Daim T (2019). Technology forecasting by analogy-based on social network analysis: The case of autonomous vehicles. Technol Forecast Soc Change.

[37] Nagarajan K, Muniyandi M, Palani B, Sellappan S (2020). Social network analysis methods for exploring SARS-CoV-2 contact tracing data. BMC Med Res Methodol.

[38] Das K, Samanta S, Pal M (2018). Study on centrality measures in social networks: a survey. Soc Netw Anal Min.

[39] Hwang JG, Ko YS, Lee C, Hwang JS (2019). Analysis results in technical Ttends of 2018 Farnborough International Airshow via centrality analysis. J Korea Acad-Industr Coop Soc.

[40] Pomare C, Long JC, Churruca K, Ellis LA, Braithwaite J (2022). Social network research in health care settings: design and data collection. Soc Netw.

